# LC-MS/MS versus TLC plus GC methods: Consistency of glycerolipid and fatty acid profiles in microalgae and higher plant cells and effect of a nitrogen starvation

**DOI:** 10.1371/journal.pone.0182423

**Published:** 2017-08-03

**Authors:** Juliette Jouhet, Josselin Lupette, Olivier Clerc, Leonardo Magneschi, Mariette Bedhomme, Séverine Collin, Sylvaine Roy, Eric Maréchal, Fabrice Rébeillé

**Affiliations:** Laboratoire de Physiologie Cellulaire Végétale, Unité mixte de recherche 5168 CNRS - CEA - INRA - Université Grenoble Alpes, Bioscience and Biotechnologies Institute of Grenoble, CEA Grenoble, Grenoble, France; National Taiwan University, TAIWAN

## Abstract

Methods to analyze lipidomes have considerably evolved, more and more based on mass spectrometry technics (LC-MS/MS). However, accurate quantifications using these methods require ^13^C-labeled standards for each lipid, which is not feasible because of the very large number of molecules. Thus, quantifications rely on standard molecules representative of a whole class of lipids, which might lead to false estimations of some molecular species. Here, we determined and compared glycerolipid distributions from three different types of cells, two microalgae (*Phaeodactylum tricornutum*, *Nannochloropsis gaditana*) and one higher plant (*Arabidopsis thaliana*), using either LC-MS/MS or Thin Layer Chromatography coupled with Gas Chromatography (TLC-GC), this last approach relying on the precise quantification of the fatty acids present in each glycerolipid class. Our results showed that the glycerolipid distribution was significantly different depending on the method used. How can one reconcile these two analytical methods? Here we propose that the possible bias with MS data can be circumvented by systematically running in tandem with the sample to be analyzed a lipid extract from a qualified control (QC) of each type of cells, previously analyzed by TLC-GC, and used as an external standard to quantify the MS results. As a case study, we applied this method to compare the impact of a nitrogen deficiency on the three types of cells.

## Introduction

Understanding lipid metabolism is today an important field of research with potential applications in various domains such as health, food, biofuel, green chemistry and many others [1, 2]. In this context, the number of reports concerning the characterization of lipidomes from numerous varieties of plants and microalgae increased exponentially this past decade. Microalgae, in particular those present in marine phytoplankton, are responsible for up to one-fourth of global primary productivity [3], and are also potential sources for next generation biofuel. An emerging field of research involves metabolic engineering of various strains of microalgae to increase lipid synthesis and accumulation [4–6]. Renewable biofuels are produced from triacylglycerols (TAG), a neutral lipid that accumulates in lipid droplets. TAG are triesters of fatty acids (FA) and glycerol, and are members of the glycerolipid family which also includes most of the polar membrane lipids. Membrane glycerolipids are diesters of fatty acids (FA) and glycerol, the third hydroxyl function of the glycerol backbone being linked with a polar group [7, 8]. Several pathways can be involved in the synthesis of TAG which are produced in the endoplasmic reticulum (ER) by acylation at the sn-3 position of diacylglycerol (DAG), a reaction catalyzed by an acyl-coA dependent DAG acyltransferase [1]. DAG can originate from de novo synthesis or by the recycling of membrane lipids [9, 10]. Another route to produce TAG involves a phospholipid:DAG acyltransferase (PDAT) activity [9], where the FA moiety added at the sn-3 position of the glycerol backbone originates from a membrane lipid (mostly phosphatidylcholine, PC), and not from the acyl-CoA pool [11]. Thus, membrane and storage lipids are intimately connected, and it is required to have a good understanding of the metabolisms and regulations involved in TAG synthesis to have a complete overview of the glycerolipidome.

Glycerolipids can be categorized in different classes depending on the polar head, but in each of these classes they may also differ by the nature of their FA, thus leading to a very high number of different molecules [7]. Methods for routine analyses of glycerolipids have considerably evolved. Although the techniques for lipid extraction remain almost unchanged, based on methods initially developed by Bligh and Dyer [12] or Folch [13], the techniques for quantification are remarkably renewed. Up to recently, classical methods were based on the separation of the various glycerolipid classes by thin layer chromatography (TLC) followed by the extraction from the TLC of lipids present in each classes, then methanolysis of the associated FA to form methyl ester of FA (FAME). FAME were then determined and quantified by gas chromatography coupled to flame ionization detector (GC-FID) [14, 15]. These methods are robust and have not become obsolete as they allow the absolute quantification of glycerolipids through their FA based on the response of the FID detector that is linear for a wide range of FA concentrations and also for a broad range of FA chain lengths [16]. However, these methods do not allow to determine the FA composition in each lipid molecule (i.e. the FA present at *sn-1* and *sn-2* positions of the glycerol backbone). In addition, they are time-consuming and not well adapted to high throughput analyses of lipidomes from engineered strains. For these reasons, new methods have been developed based on electrospray ionization (ESI) of the lipid extracts coupled with mass spectrometry (MS) analyses [17]. Indeed, electrospray tandem mass spectrometry (ESI-MS/MS) offers an attractive alternative for the determination of glycerolipids because of its sensitivity and specificity [18]. With this method, molecules are first softly ionized in the ESI source, with very little fragmentation. These ions are then injected in the tandem MS/MS where they are isolated then fragmented by collision with an inert gas, the masses resulting from the fragmented ions being ultimately determined [19, 20]. Because the way the glycerolipid molecules fragment depends on the class of the lipid, it is theoretically possible to identify the initial (parent) ion introduced in the MS analyzer [19, 21]. Quantification is however difficult because the yield of ionization and ultimately the response of the MS analyzer depends on several parameters including the nature of the molecule to be analyzed and the chemical environment of this molecule. Indeed, the molar response can vary from one class of lipid to another, and also between homologs within a class depending on the mass of the molecule (i.e. the nature of the FA attached to the glycerol backbone) and energy collision [18, 20].

To correct these biases, internal standards are required. The ideal internal standard has the same structure as the molecule to be analyzed, but is labeled with ^13^C to be differentiated by its mass from the native molecule. Because of the very high number of molecules that need to be determined, it is not feasible to have ^13^C-labeled internal standards corresponding to each of them. Instead, internal standards representing a class of lipids but with masses not found in the biological samples are used, for example glycerolipids containing saturated FA. Previous works indicated that internal standard cocktail with at least two internal standards for each class [17, 22], and also complex adjustment factors [23], are required for quantification. However, these adjustment factors can vary with the type of ESI-MS/MS apparatus used for the analyses or may change with time depending on the calibration parameters of the spectrometer. Furthermore, it is not always possible to find commercially available internal standards for all glycerolipid classes. At the end, and without a complete set of authentic ^13^C-labeled standards, absolute quantification of lipids by ESI-MS/MS is difficult, and fold-change is likely to be the best way to present the data when comparing different strains from same species. However, it is also important to know the relative amount of each glycerolipid present in one given sample, for example the MGDG/PC ratio which reflects the plastid membrane network in the cell [8], and this might be biased with the ESI-MS/MS method. Furthermore, absolute quantifications such as those obtained with TLC plus GC-FID are required to estimate the yield of productivity of engineered strains and to estimate how is affected the quantity of each lipid class [24].

In the present article, we compared the glycerolipid profiles obtained by either LC-MS or TLC plus GC-FID methods from two strains of microalgae, *Nannochloropsis gaditana* and *Phaeodactylum tricornutum*, and one higher plant cell culture, *Arabidopsis thaliana*. The results obtained with the two analytical techniques appeared significantly different. To correct the biases associated with the LC-MS method, we introduced correcting factors based on the results obtained with the GC-FID technique. This quantification method is based on the analysis of a lipid extract from a qualified control (QC), quantified once by TLC plus GC-FID, and then systematically run with the samples to be analyzed by ESI-MS/MS. This method takes advantage of the sensitivity and speed of the ESI-MS/MS methods, and allows profiling and quantification of glycerolipids that are directly comparable with those obtained with the TLC plus GC-FID techniques. We used this method to quantify the effect of a nitrogen deprivation on the three different types of cells.

## Material and methods

### Cell cultures

*Phaeodactylum tricornutum* Pt1 was obtained from the Culture Collection of Algae and Protozoa (CCAP 1055/3). Cells were maintained and grown in Enriched Seawater Artificial Water (ESAW) medium, as described [25, 26]. Cells were grown in the presence of 0.554 mM of nitrogen (N) and 0.0224 mM of phosphorus (P) (ESAW 10N10P). For Qualified Control (QC) preparations, cultures were grown in exponential phase in two times 2 liters flasks with 100 rpm shaking, an irradiance of 100 μE and a 12 hours light / 12 hours dark photoperiod at 20°C. Cells were collected by centrifugation (3500 rpm during 15 minutes at 4°C) after 12 days. The pellets were cryopreserved in liquid nitrogen and frozen at -80°C. The concentration of *Phaeodactylum tricornutum* was determined with a Tecan Infinite ^®^ M1000 PRO. For nitrogen deficiency experiment, cells were seeded at 1x10^6^ cells/mL in 50 mL of ESAW 10N10P or 0N10P (in which NaNO_3_ was omitted) and grown for 7 days under a 12:12 light (60 μE.m^-2^.sec^-1^) / dark cycle at 20°C, 250 rpm shaking speed.

*Nannochloropsis gaditana* Lubian Strain CCMP526 (Culture Collection of Marine Phytoplankton, now known as NCMA: National Center for Marine Algae and Microbiota) was used in all experiments. For Qualified Control preparation (QC), cells were grown in small photobioreactors (Multi-Cultivator MC 1000, Photon Systems Instruments, Czech Republic). Cell pre-cultures were centrifuged at 3500g for 5 minutes and re-suspended in medium E [27] to a final concentration of 2·10^6^ cells/ml. Growth was constantly monitored via optical densities in presence of a constant light regime at 20°C; culture mixing throughout cultivation time was provided by gas sparging in which CO_2_ levels were maintained constant at 0.5% as in air-lift photobioreactors. Precise and constant CO_2_ supplies to bioreactor tubes were provided by the Gas Mixing System GMS 150 (Photon Systems Instruments, Czech Republic) following manufacturer’s instructions. After 7 days of growth, the entire amount of culture was harvested and stored in liquid nitrogen for lipid extraction. For nitrogen deficiency experiment, cells were seeded at 2.5·10^6^ cells/mL in 50 mL of either nitrogen-replete ESAW10N10P or nitrogen-free ESAW0N10P, in which NaNO_3_ was omitted. Cultures were grown under a 12:12 light (60 μE.m^-2^.sec^-1^) / dark cycle at 20°C, 250 rpm shaking speed and samples for lipid analysis and determination of cell concentrations were taken at day 7 from the beginning of the experiment. For accuracy, cells were counted using a LUNA^™^ Automated Cell Counter following manufacturer's instructions.

*Arabidopsis thaliana* cell cultures were maintained in 200 mL of MS medium (MSP09, Caisson Laboratories, Inc, USA) containing 4 mM of phosphate, 1.5% (w/v) sucrose and 1.2 mg.L^-1^ 2,4-dichlorophenoxyacetic acid, as described previously [28]. Cultures were kept under continuous light (100 μE.m^-2^.s^-1^) at 22°C, and agitated with rotary shaking at 125 rpm and subcultured every 7 days. For the preparation of the Arabidopsis QC lipid extract, 4 flasks corresponding to 18 g of fresh weight of Arabidopsis cells were harvested 3 days after renewing the media. For nitrogen deficiency experiments, the cells were washed 3 times in a MS medium deprived of nitrogen (MSP09-50LT, Caisson Laboratories, Inc, USA) and grown in the same nitrogen-deprived medium for 72h.

### Glycerolipid extraction

Glycerolipids were extracted from freeze-dried cells. First, cells were harvested by centrifugation for *Phaeodactylum tricornutum* and *Nannochloropsis gaditana* or by filtration for *Arabidopsis thaliana* and then immediately frozen in liquid nitrogen. Once freeze-dried, the pellet was suspended in 4 mL of boiling ethanol for 5 minutes to prevent lipid degradation and lipids were extracted according to Folch [13] by addition of 2 mL methanol and 8 mL chloroform at room temperature. The mixture was then saturated with argon and stirred for 1 hour at room temperature. After filtration through glass wool, cell remains were rinsed with 3 mL chloroform/methanol 2:1, v/v and 5 mL of NaCl 1% were then added to the filtrate to initiate biphase formation. The chloroform phase was dried under argon before solubilizing the lipid extract in pure chloroform. For QC lipid extract, because of the higher quantity of starting material, volumes for extraction were multiplied by 10.

### TLC and GC-FID lipid quantification

Total glycerolipids were quantified from their fatty acids: in an aliquot fraction, 5 μg of 15:0 (internal standard) were added and the fatty acids present were converted to methyl esters (FAME) by a 1-hour incubation in 3 mL 2.5% H_2_SO_4_ in pure methanol at 100°C [29]. The reaction was stopped by addition of 3 mL water and 3 mL hexane. The hexane phase was analyzed by gas chromatography-flame ionization detector (GC-FID) (Perkin Elmer) on a BPX70 (SGE) column. FAMEs were identified by comparison of their retention times with those of standards (Sigma) and quantified using 15:0 for calibration. Extraction and quantification were performed at least 3 times. To quantify the various classes of neutral and polar glycerolipids, 300 μg of lipids were separated by thin layer chromatography (TLC) onto glass-backed silica gel plates (Merck) using two distinct resolving systems [30]. To isolate neutral lipids including DAG and TAG, lipids were resolved by TLC run in one dimension with hexane:diethylether:acetic acid (70:30:1, v/v). To isolate membrane glycerolipids, lipids were resolved by two-dimensional TLC. The first solvent was chloroform:methanol:water (65:25:4, v/v) and the second one chloroform:acetone:methanol:acetic acid:water (50:20:10:10:5, v/v). Lipids were then visualized under UV light, after spraying with 2% 8-anilino-1-naphthalenesulfonic acid in methanol, and scraped off the plate. Lipids were quantified by methanolysis and GC-FID directly from the scraped silica as described above.

### Determination of sn-1, sn-2 positions by ion trap MS

The position of the FA esterified to the glycerol backbone of the various glycerolipids was determined based on ion trap MS analyses. Glycerol carbons were numbered following the stereospecific number (*sn*) nomenclature. Depending on the nature of the glycerolipid and the type of adduct, the substituents at *sn-1* (or *sn-3*) and *sn-2* positions are differently cleaved when submitted to low energy collision-induced dissociation [14]. This is reflected in MS analyses by the preferential loss of one of the two fatty acids, leading to a dissymmetrical abundance of the collision fragments.

For ion trap MS analyses, purified lipid classes were dissolved in 10 mM ammonium acetate in pure methanol. They were introduced by direct infusion (ESI-MS) into a trap type mass spectrometer (LTQ-XL, Thermo Scientific) and identified by comparison with standards. In these conditions, the produced ions were mainly present as H^-^, H^+^, NH_4_^+^ or Na^+^ adducts. Lipids were identified by MS analysis with their precursor ion or by neutral loss analyses after low energy collision-induced dissociation, as previously described [14]. FA positions on the glycerol backbone were determined by the properties of the MS2 fragments as summarized here (Table 1), and previously described [14]. Briefly, glycerol carbons were numbered following the stereospecific number (*sn*) nomenclature. Depending on the nature of the glycerolipid and the type of adduct, the FA at *sn-1* (or *sn-3*) and *sn-2* positions are differently cleaved when submitted to low energy collision-induced dissociation. This is reflected in MS/MS analyses by the preferential loss of one of the two fatty acids, leading to a dissymmetrical abundance of the collision fragments. The patterns of MS2 fragments for all glycerolipids have been described in previous studies (Table 1), except for diacylglyceryl-hydroxymethyl-N,N,N-trimethyl-β-alanine (DGTA). In the present study we hypothesized that the loss of fatty acids in DGTA following low energy collision-induced dissociation are similar to those observed for other polar lipids, such as phosphatidylcholine (PC). All experiments were made in triplicate.

**Table 1:** Conditions for the regiochemical assignment of fatty acids at *sn*-1, *sn*-2 and *sn*-3 positions in glycerolipids.

Analyzed lipids	Parental ion to be fragmented	MS2 fragments properties	References
**Phospholipids**			
phosphatidylcholines (PC)	[M+H]^+^	[M+H-R_2_CH = C = O]^+^>[M+H-R_1_CH = C = O]^+^	[31]
Phosphatidylethanolamines (PE)	[M-H]^-^	[R_2_COO]^-^>[R_1_COO]^-^	[32]
phosphatidylglycerols (PG)	[M-H]^-^	[M-H-R_2_COOH]^-^>[M-H-R_1_COOH]^-^	[33]
phosphatidylinositols (PI)	[M-H]^-^	[M-H-R_2_COOH]^-^>[M-H-R_1_COOH]^-^	[34]
Phosphatidic acids (PA)	[M-H]^-^	[M-H-R_2_COOH]^-^>[M-H-R_1_COOH]^-^	[35]
Cardiolipins (DPG)	[M-H]^-^	MS2 produced the corresponding PA	[36]
**Non phosphorus glycerolipids**			
sulfoquinovosyldiacylglycerols (SQDG)	[M-H]^-^	[M-H-R_1_COOH]^-^>[M-H-R_2_COOH]^-^	[37]
Monogalactosyldiacylglycerols (MGDG)	[M+Na]^+^	[M+Na-R_1_COO^-^]^+^>[M+Na-R_2_COO^-^]^+^	[38]
digalactosyldiacylglycerols (DGDG)	[M+Na]^+^	[M+Na-R_1_COO^-^]^+^>[M+Na -R_2_COO^-^]^+^	[38]
diacylglyceryl-hydroxymethyl-N,N,N-trimethyl-β-alanine (DGTA)	[M+H]^+^	[M+H-R_2_COOH]^+^>[M+H-R_1_COOH]^+^	By analogy with phospholipid diacylglycerol moiety
diacylglyceryl-hydroxymethyl-N,N,N-trimethyl-homoserine (DGTS)	[M+H]^+^	[M+H-R_2_COOH]^+^>[M+H-R_1_COOH]^+^ Can be differentiated from DGTA by a neutral loss of *m/z* 87	By analogy with phospholipid diacylglycerol moiety
**Neutral lipids**			
Diacylglycerol (DAG)	[M+NH_4_]^+^	[M+NH_4_-R_1_COONH_4_]^+^>[M+NH_4_-R_2_COONH_4_]^+^	[39]
Triacylglycerol (TAG)	[M+NH_4_]^+^	[M+NH_4_-R_1/3_COO^-^]^+^>[M+ NH_4_-R_2_COO^-^]^+^	[40]

### HPLC and MS/MS analyses

The lipid extracts corresponding to 25 nmol of total FA were dissolved in 100 μL of chloroform/methanol [2/1, (v/v)] containing 125 pmol of each internal standard. Internal standards were obtained from Avanti Polar Lipids Inc. for PC 18:0–18:0, PE 18:0–18:0, PI 18:0–18:0, PS 18:0–18:0, PG 18:0–18:0, PA 18:0–18:0, DGTS 16:0–16:0, DAG 18:0–22:6, TAG 18:0–18:0–18:0 and DPG14:0–14:0–14:0–14:0, or synthesized [41, 42] for MGDG 18:0–18:0 and DGDG 16:0–16:0, or extracted from spinach thylakoid [43] and hydrogenated as described [44] for SQDG 16:0–18:0. All the internal standard solutions were first quantified by GC-FID. For the MS/MS-QC method, only DAG, PE and SQDG were used as internal standard representing the three chromatographic segments (S1 Table). DAG 18:0–22:6 is used to adjust DAG, TAG, MGDG and DGDG amounts, PE 18:0–18:0 for PE amount and SQDG 16:0–18:0 for SQDG, DGTA, DGTS, PA, PC, PG, PS, DPG and PI amounts. In addition, the QC lipid extract was run in the middle of the sample list within each series of analyses. These QC extracts corresponded to a known lipid extract from *Phaeodactylum tricornutum*, *Nannochloropsis gaditana* or *Arabidopsis thaliana* cell cultures qualified and quantified by TLC and GC-FID as described by [14]. Lipids were then separated by HPLC and quantified by ESI-MS/MS.

The HPLC separation method was adapted from [45]. Lipid classes were separated using an Agilent 1200 HPLC system using a 150 mm×3 mm (length × internal diameter) 5 μm diol column (Macherey-Nagel), at 40°C. The mobile phases consisted of hexane/isopropanol/water/ammonium acetate 1M, pH5.3 [625/350/24/1, (v/v/v/v)] (A) and isopropanol/water/ammonium acetate 1M, pH5.3 [850/149/1, (v/v/v)] (B). The injection volume was 20 μL, corresponding to 5 nmol of total fatty acid, and each sample was injected 3 times as technical replicates (relative standard deviation (RSD) < 5% for each lipid class). We verify that the method is within a linear range up to 15 nmol of total fatty acid (S1 Fig). After 5 min, the percentage of B was increased linearly from 0% to 100% in 30 min and stayed at 100% for 15 min. This elution sequence was followed by a return to 100% A in 5 min and an equilibration for 20 min with 100% A before the next injection, leading to a total runtime of 70 min. The flow rate of the mobile phase was 200 μL/min. The distinct glycerophospholipid classes were eluted successively as a function of the polar head group. Under these conditions, they were eluted in the following order: TAG, DAG, MGDG, DGDG, PE, DGTS, DGTA, PG, PI, SQDG, PS, PC, DPG and PA.

**Fig 1:**
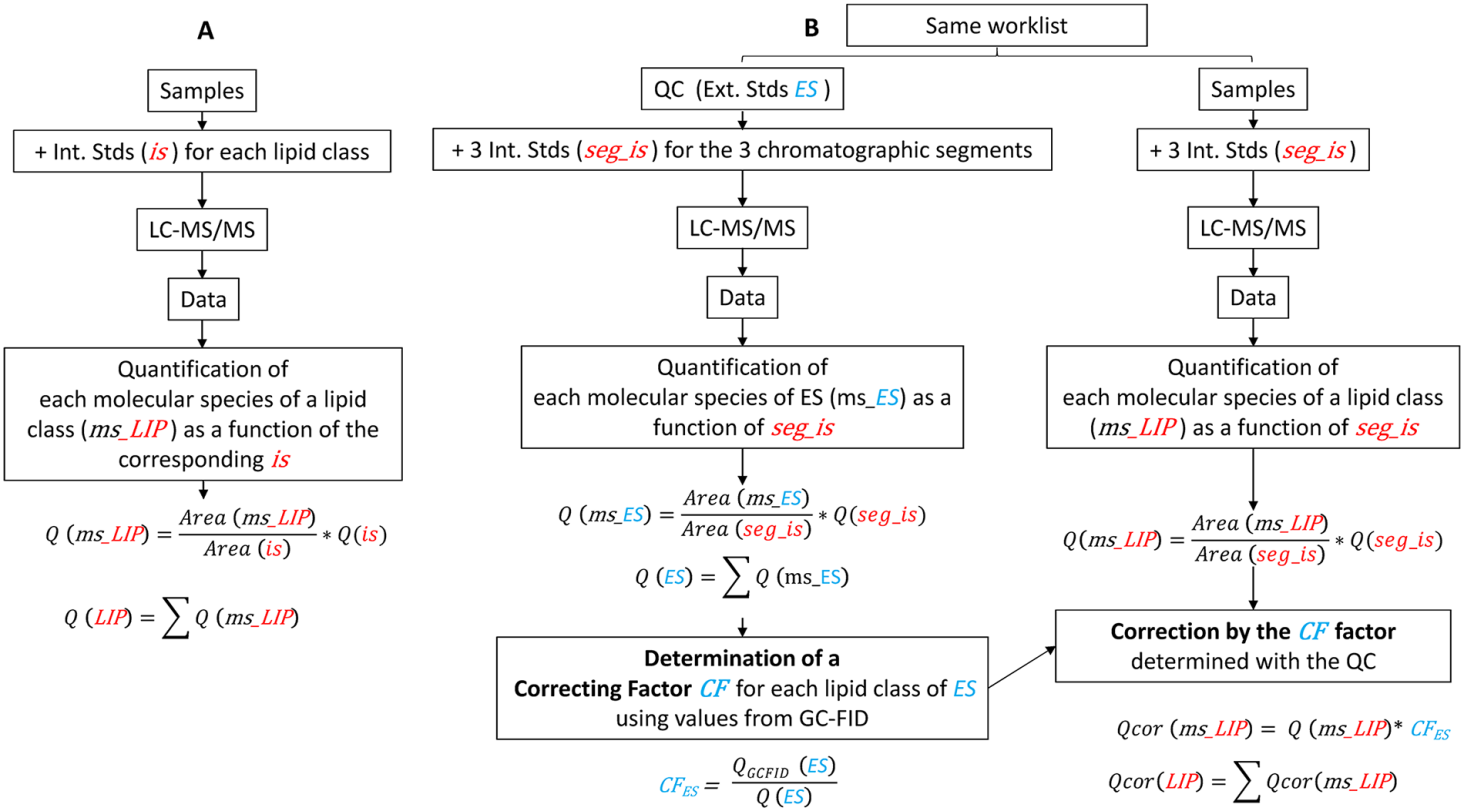
Diagram representing our strategies to quantify glycerolipids by LC-MS/MS. A) In MS/MS-Internal Stds method, one internal standard is used per lipid class. B) In MS/MS-QC method, one internal standard is used for each chromatographic segment: DAG 18:0–22:6 is used to adjust DAG, TAG, MGDG and DGDG amounts, PE 18:0–18:0 for PE amount and SQDG 16:0–18:0 for SQDG, DGTA, DGTS, PA, PC, PG, PS, DPG and PI amounts.

Mass spectrometric analysis was done on a 6460 triple quadrupole mass spectrometer (Agilent) equipped with a Jet stream electrospray ion source under following settings: Drying gas heater: 260°C, Drying gas flow 13 L/min, Sheath gas heater: 300°C, Sheath gas flow: 11L/min, Nebulizer pressure: 25 psi, Capillary voltage: ± 5000 V, Nozzle voltage ± 1000. Nitrogen was used as collision gas. The quadrupoles Q1 and Q3 were operated at widest and unit resolution respectively. Specific MRM scans used to define molecular species are summarized in S1 Table. Mass spectra were processed by MassHunter Workstation software (Agilent). For the MS/MS-Internal Stds method, each class of lipids (in the pmol range) was quantified with the corresponding internal standard. For the MS/MS-QC method, a QC sample is used as an external standard, and run with the list of the samples to be analyzed. First, lipid amounts in all samples were adjusted with three internal standards (see above) to correct possible variations linked to the injection and analytical run. Then, within the QC samples, molecules in a given class of glycerolipid were summed and compared to the amount of the same lipid class previously determined by TLC-GC-FID. This is done in order to establish a correspondence between the area of the peaks and a number of pmoles. These corresponding factors were then applied to the samples of the list to be analyzed (Fig 1).

## Results and discussion

### Determinations of glycerolipid and FA profiles by TLC plus GC-FID or LC-MS/MS methods

**Fig 2:**
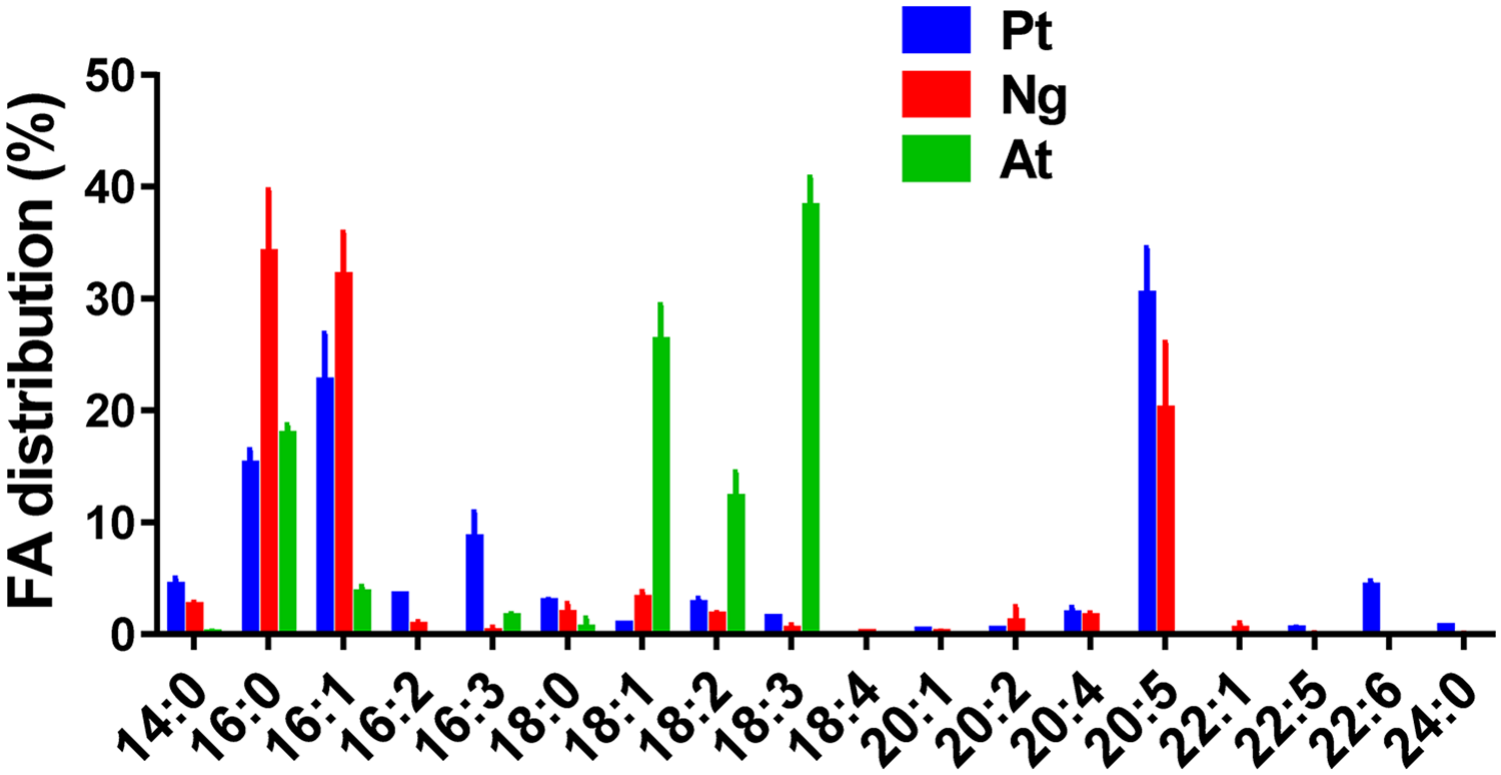
FA distribution in *Phaeodactylum tricornutum* (P.t.), *Nannochloropsis gaditana* (N.g.) and *Arabidopsis thaliana* (A.t.) cell cultures. Lipids were extracted and FA were converted to FAME before GC-FID analyses, as described in Material and Methods. Values are the average ± SD of three biological repeats for each type of cells.

GC-FID allows the absolute quantification of FA because FID signals are directly proportional to the total hydrocarbon content flowing through the detector [16]. This is not the case with ESI-MS/MS because the yield of electrospray ionization depends on the injected molecule and also on the technology of the ESI source, which varies from one manufacturer to another. Thus, careful and specific optimization of the LC protocol and MS conditions are required for the compounds that do not have matched internal standards, i.e. for most of the glycerolipids we need to analyze. This is not easily compatible with high throughput analyses where, ideally, all the compounds have to be quantified in one run. In a first approach, we developed a LC protocol to separate in one run most of the glycerolipids (see Material and methods), and then we quantified these lipids by ESI-MS/MS. In a second step, we compared these results with those obtained after separation of the lipids by TLC followed by the analyses of their FA by GC-FID. To compare TLC plus GC-FID and LC-MS/MS methods, we used three strains of cells having very different FA profiles. As shown in Fig 2, the diatom *Phaeodactylum tricornutum* (P.t.) and the Eustigmatophyte *Nannochloropsis gaditana* (N.g.) display a total FA profile poor in 18C but rich in 16C (16:0 and 16:1) and long polyunsaturated FA chain (20:5), whereas the FA profile of *Arabidopsis thaliana* (A.t.) cell cultures is rich in 18C and shows almost no FA with a number of C > 18.

**Fig 3:**
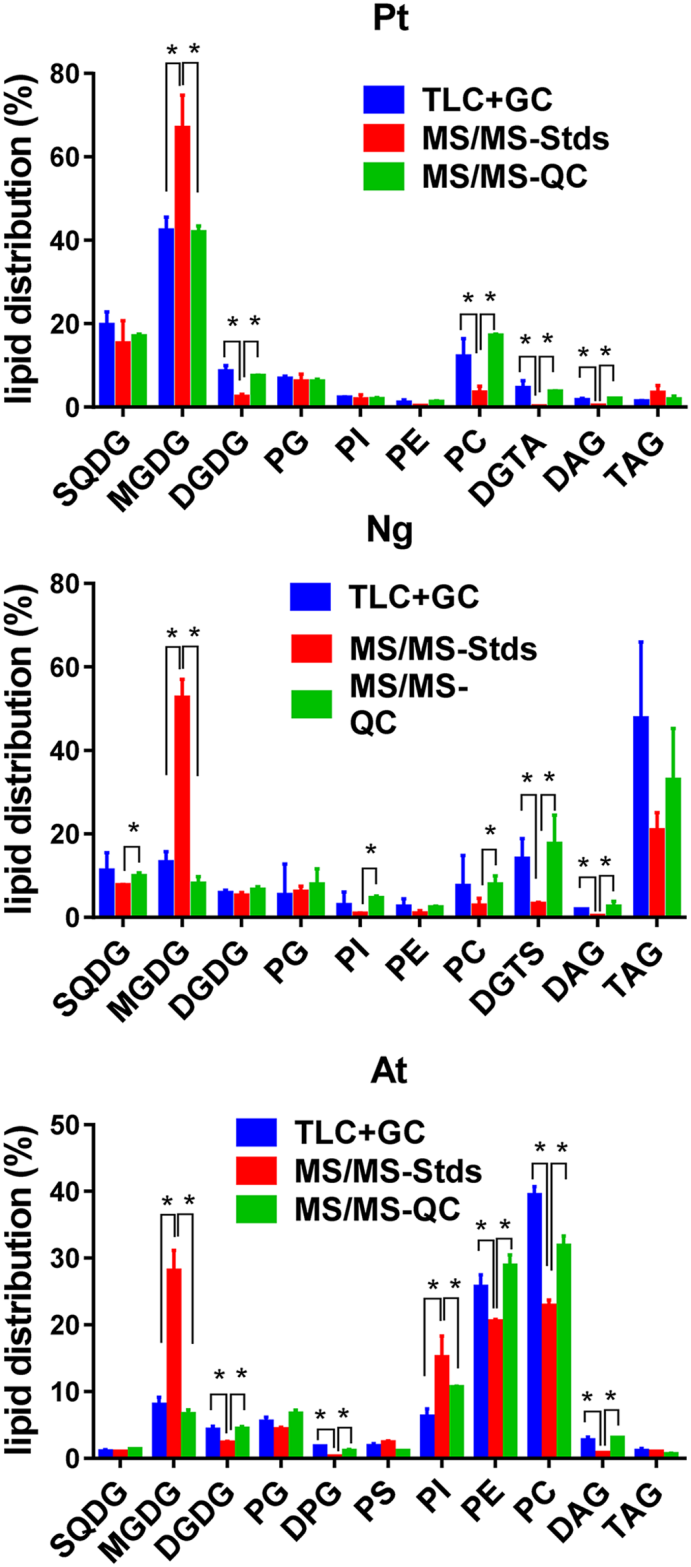
Lipid distribution in P.t., N.g. and A.t.. Lipids were extracted and each class of lipids was quantified either by TLC plus GC-FID (TLC+GC bars), or by LC-MS/MS with an internal standard for each class (MS/MS-Stds bars), or by LC-MS/MS using a QC extract run in tandem with the sample (MS/MS-QC bars) (see Material and methods). Values are the average ± SD of three biological repeats for each type of cells. Values are the average ± SD of three biological repeats. Significant differences (P < 0.05) are shown by an asterisk and were calculated by an unpaired multiple t test using GraphPad Prism software.

To identify and quantify the main glycerolipid molecules by ESI-MS/MS, it is convenient in a first step not only to determine the mass of each of them, but also to identify the nature of the FA attached to the glycerol backbone. Thus, a limited number of Multiple Reaction Monitoring (MRM) transitions can be analyzed, saving time and providing better accuracy in the quantification of each ion. This also allows to determine the FA profile for each class of glycerolipids, which could be thereafter compared to the FA profile established by the TLC plus GC-FID method. This was done with N.g. and A.t. cells as previously described for P.t. [14]. Glycerolipids were first separated by TLC and then scraped off the plates for analysis into a trap-type mass spectrometer. The methods used for the identification of main glycerolipids and to determine the nature and positioning of FA were as reported previously [14] for P.t. (see also Table 1), and the results are shown in S2 and S3 Tables for N.g. and A.t., respectively. Knowing the mass of each glycerolipid potentially present, the lipid extracts were thereafter analyzed by LC-MS/MS, as described in Material and Methods. For each main class of glycerolipid we used one internal standard displaying saturated FA (16:0 or18:0, except for DAG standard which is 16:0/22:6) these standards molecules being not found in the different extracts. The various masses associated with a same class of lipid were then quantified, summed up, and the results were compared with the values obtained by the TLC plus GC-FID method. As shown in Fig 3, the profiles were quite different depending on the technique that was used (TLC+GC bars versus MS/MS-Stds bars in Fig 3).

Thus, when the MS profiles were calculated using one internal standard for each class of lipid, monogalactosyldiacylglycerol (MGDG) was systematically overestimated when compared to TLC plus GC-FID, whereas digalactosyldiacylglycerol (DGDG), phosphatidylcholine (PC), DAG, diacylglyceryl-hydroxymethyl-N,N,N-trimethyl-β-alanine (DGTA) or diacylglyceryl-hydroxymethyl-N,N,N-trimethylhomoserine (DGTS) (P.t. and N.g., respectively) were underestimated. If we assume that GC-FID is the method of reference for the glycerolipid quantification, then these results indicate that there are classes of glycerolipids that cannot be precisely quantified by our LC-MS/MS protocol, probably due to some matrix effect or because the nature of the FA could possibly impact on the ionization efficiency in the ESI source, as already reported [23]. The quantification also did not match the GC-FID measurements. Indeed, recovery experiments indicate that the quantifications based on the presence of internal standards for each class of lipids were generally overestimating the total amount of nmoles injected in the LC (S1A Fig, MS/MS-Stds curves). Distribution and quantification of glycerolipids are important parameters when studying membrane remodeling, such as in the case of Pi deficiency where PC in non-photosynthetic membranes is replaced by MGDG [46]. Also, it remains important to be able to compare recent and older results, whatever the technology used.

**Fig 4:**
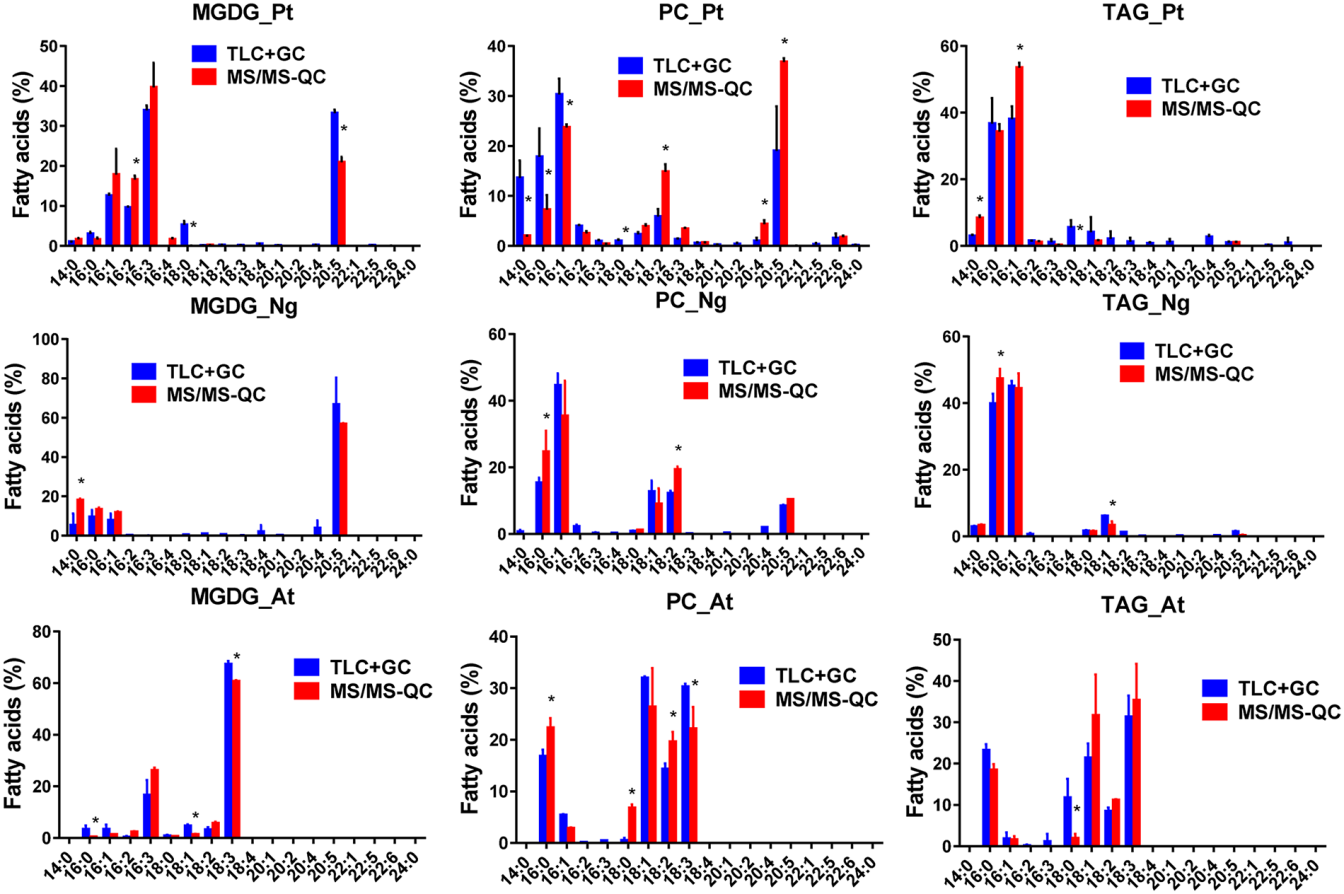
FA distribution in three main classes of lipids extracted from P.t., N.g. and A.t.. The distribution was calculated using either the TLC plus GC-FID or the MS/MS-QC methods. Whereas the TLC plus GC method give directly the FA composition present in a given class of glycerolipid, the FA composition needs to be calculated with the LC-MS/MS approach knowing the proportion of each species present in the class and the nature of the FA attached to the glycerol backbone. Values are the average ± SD of three biological repeats. Significant differences (P < 0.05) for main FA are shown by an asterisk and were calculated by an unpaired multiple t test using GraphPad Prism software.

To work around this problem, and with the idea to keep the method as straightforward as possible, we developed a method based on qualified controls. In this method, a lipid extract from a large batch of the type of cells to be analyzed is first quantified for its glycerolipid content by the TLC plus GC-FID method. This is done once, and then this same lipid extract will serve as a qualified control (QC) for all the experiments using these cells. In other words, the lipids from this QC will be used as external standards. Thus, when glycerolipids have to be quantified by ESI-MS/MS, a fraction of the QC is also analyzed in the same series of samples. Then, following ESI-MS/MS analyses, molecules of the QC belonging to a given class of glycerolipid are summed and compared to the amount of the same lipid class previously determined by TLC plus GC-FID. This allows to establish a correspondence between the area of the peaks and a number of pmoles. The correspondence factors are thereafter applied to the samples of interest (Fig 1). Because the lipid extract from the QC has been prepared in the same way as the lipid extract from the sample, the yield of ionization, if affected by the protocol of extraction, should be the same in both cases. In the QC, as well as in the sample of interest, three internal standards were added to correct possible variations during the injection and LC separation processes. The internal standards 18:0/22:6 DAG, 18:0/18:0 phosphatidylethanolamine (PE) and 18:0/18:0 sulfoquinovosyldiacylglycerol (SQDG), were chosen for their LC retention times in order to cover the whole separation step preceding the ESI-MS/MS analyses. As shown in Fig 3, this method allowed a better quantification of the various lipid classes, the new profiles (MS/MS-QC bars) fitting those obtained by TLC plus GC-FID (TLC+GC bars). In addition, we verified for the three types of cells that there was a good recovery of the total number of nmoles injected in the LC-MS/MS, up to 15 nmoles (S1A Fig (MS/MS-QC curves) and S1B Fig). It is interesting to note that we barely detected phosphatidic acid (PA). Although PA was present in very low amounts in P.t. and N.g. and could not be detected using the TLC approach [14, 30], this is not the case in A.t. where it represents 2–4% of all glycerolipids [15]. However, PA could not be accurately and reproducibly measured by LC-MS/MS in A.t., indicating that ionization of this glycerolipid was probably very poor. To have a better insight of potential biases linked to the nature of FA, we used the two approaches to compare the FA profiles of various representative glycerolipids. The TLC plus GC-FID method allows a direct access to the FA profile of a given class, whereas the FA profile must be reconstructed with the LC-MS/MS technique, knowing the proportion of each molecule constituting this class and the nature of their FA previously established with the MS linear trap. We compared in Fig 4 the FA profiles of MGDG, PC and TAG. As shown in this figure, the profiles obtained with both techniques are comparable, i.e. the major FA with one method are also the major FA with the other one.

There is one exception with PC from P.t.. Indeed, as previously shown [14], it is quite difficult from a P.t. lipid extract to unambiguously separate the PC and SQDG spots after TLC. Thus, following scraping off the plate, PC is likely to be cross-contaminated with SQDG, which is richer in 14:0 and poorer in 25:0 than PC. From this point of view, the FA profile determined with the ESI-MS/MS method would be more representative of the real composition of PC. In the other two cell lines, N.g. and A.t., PC and SQDG are well separated by TLC, possibly because their FA compositions contain lower amount of 20:5, and the two analytical methods give more similar results. However, both methods show differences when FA are present in low amounts, and minor FA (including 18:0, with one notable exception for PC from A.t.) are less represented with the ESI-MS/MS approach. It is likely that molecules present in very low amounts have not been observed and characterized through the MS linear trap, and thus their masses and corresponding MRM transitions have not been introduced within the MS/MS analytical software. It is indeed difficult to compute all the possible combinations associated with a specific class of glycerolipid without increasing too much the time required for measurements and losing the advantage of the method for high throughput screening of lipids.

### Case study of the impact of a nitrogen deficiency on the glycerolipid profiles of P.t., N.g. and A.t. cells

**Fig 5:**
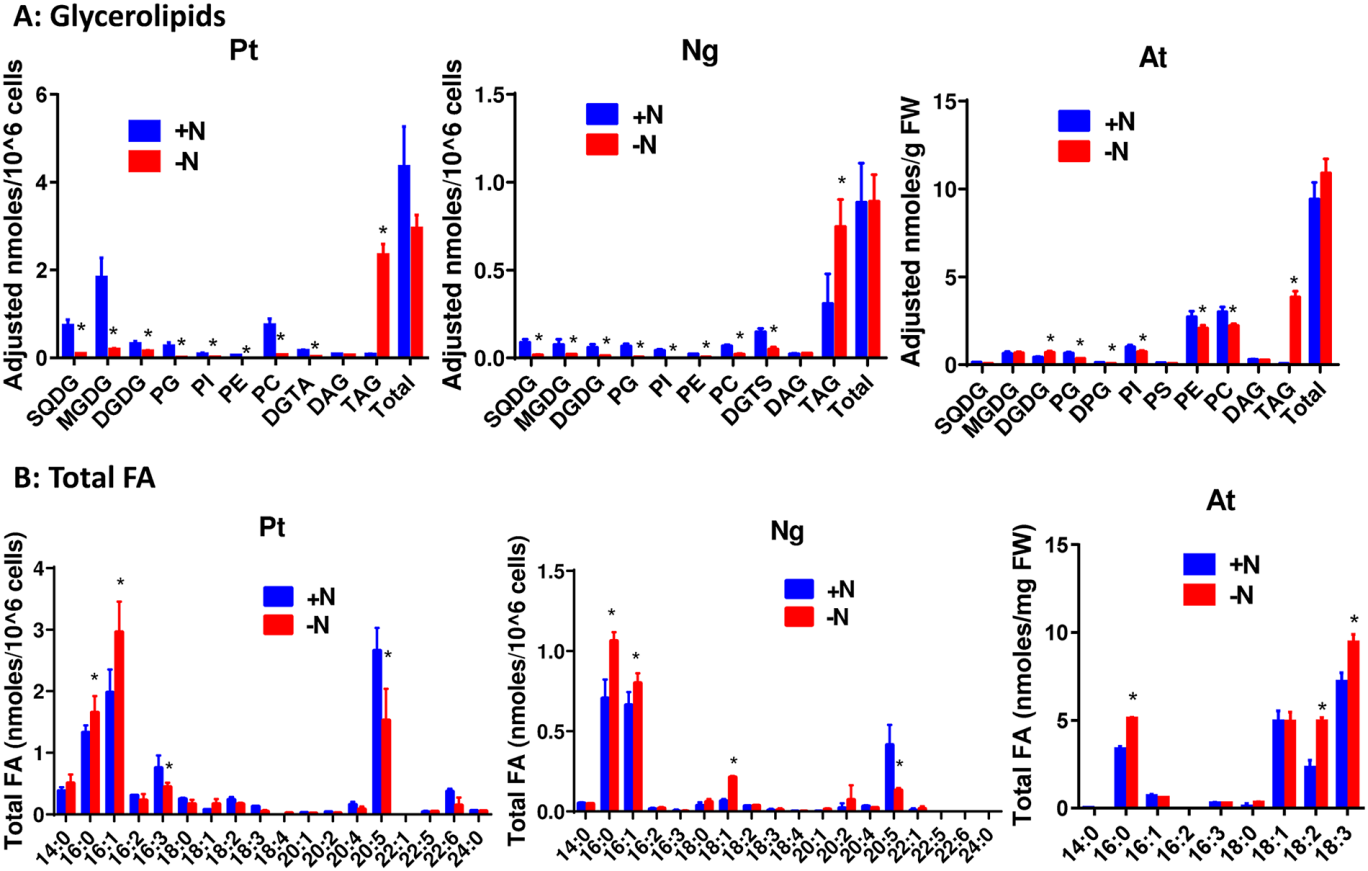
Effect of nitrogen starvation on the glycerolipid and FA composition of P.t., N.g. and A.t.. A) glycerolipid quantification using the LC-MS/MS-QC method. B) FA quantification by GC-FID. The data are the average ± SD of three biological repeats. Significant differences (p ≤ 0.05) are indicated by an asterisk. Significant differences (p < 0.05) are shown by an asterisk and were calculated by an unpaired multiple t test using GraphPad Prism software.

As a case study we used the LC-MS/MS and the QC method to quantify the impact of a nitrogen deficiency upon the glycerolipid content of the three types of cells (Fig 5).

Microalga cells were grown in a medium devoid of nitrogen for seven days, and A.t. cells were nitrogen-deprived for three days. For P.t., this corresponds to longer starvation periods than those previously reported [14]. For N.g., this study corrects a previous report lacking the analysis of DGTS [30]. As shown in this figure, TAG accumulated in the three conditions: a 30-time increase on a cell basis for P.t., a 2 to 3-time increase on a cell basis for N.g. which already displayed a fair amount of TAG in normal growth conditions, and a 60-time increase on a fresh weight basis for A.t. cells. Interestingly, the total amount of glycerolipids (polar plus neutral lipids) remained almost unchanged. In fact, in the three types of cells the accumulation of TAG was associated with a decrease of the main polar lipids, i.e. chloroplastic (SQDG, MGDG, DGDG and PG) and non chloroplastic (PC and DGTA/DGTS) in microalgae, phospholipids but not galactolipids in A.t.. In a previous experiment [14] cells were starved for a shorter period (5 days instead of 7 here) and polar lipids were less decreased, suggesting that the longer the starvation the stringer the decrease. Thus, it is likely that the decrease of polar lipids is a late event during the course of nitrogen starvation, presumably reflecting a decrease of the membrane network associated with a decrease of protein synthesis. However, the amount of DAG remained approximately constant, illustrating the important pivotal role of this molecule in the synthesis of either membrane or storage lipids. Since TAG have three FA whereas polar lipids have only two, there is in average a slight increase of the total pool of FA, but by less than a factor of 1.5, depending on the considered cells. As also shown in Fig 5, the nature of FA were impacted by nitrogen starvation. In both types of microalgae, we observed an increase of the 16C species (mainly 16:0 and 16:1) and a decrease of the long FA chains (20:5), as previously reported for P.t.. In N.g., there was also a significant increase of 18:1, whereas it was just a tendency in P.t.. In A.t., we observed an increase of 16:0 but also a shift towards polyunsaturated FA resulting from an increase of 18:2 and 18:3. This shift towards more unsaturated fatty acid species coincided with the decrease of the growth rate, as previously observed in these cells in other growth limiting conditions [28].

**Fig 6:**
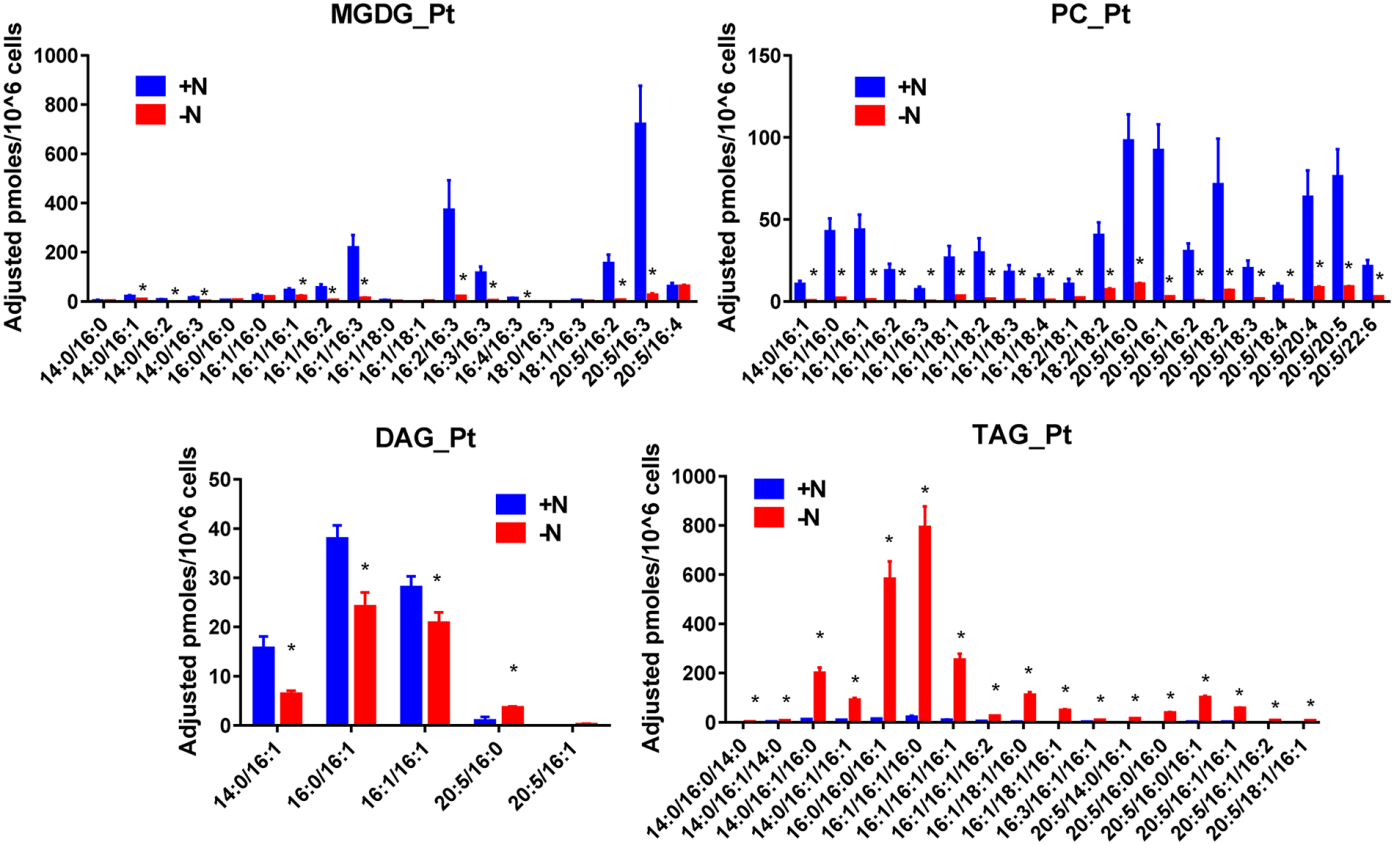
Impact of nitrogen starvation on the amount of the various molecular species constituting MGDG, PC, DAG and TAG in P.t.. Quantification were made by the LC-MS/MS method. Values are the average ± SD of three biological repeats. Significant differences (p ≤ 0.05) are indicated by an asterisk. Significant differences (P < 0.05) are shown by an asterisk and were calculated by an unpaired multiple t test using GraphPad Prism software.

**Fig 7:**
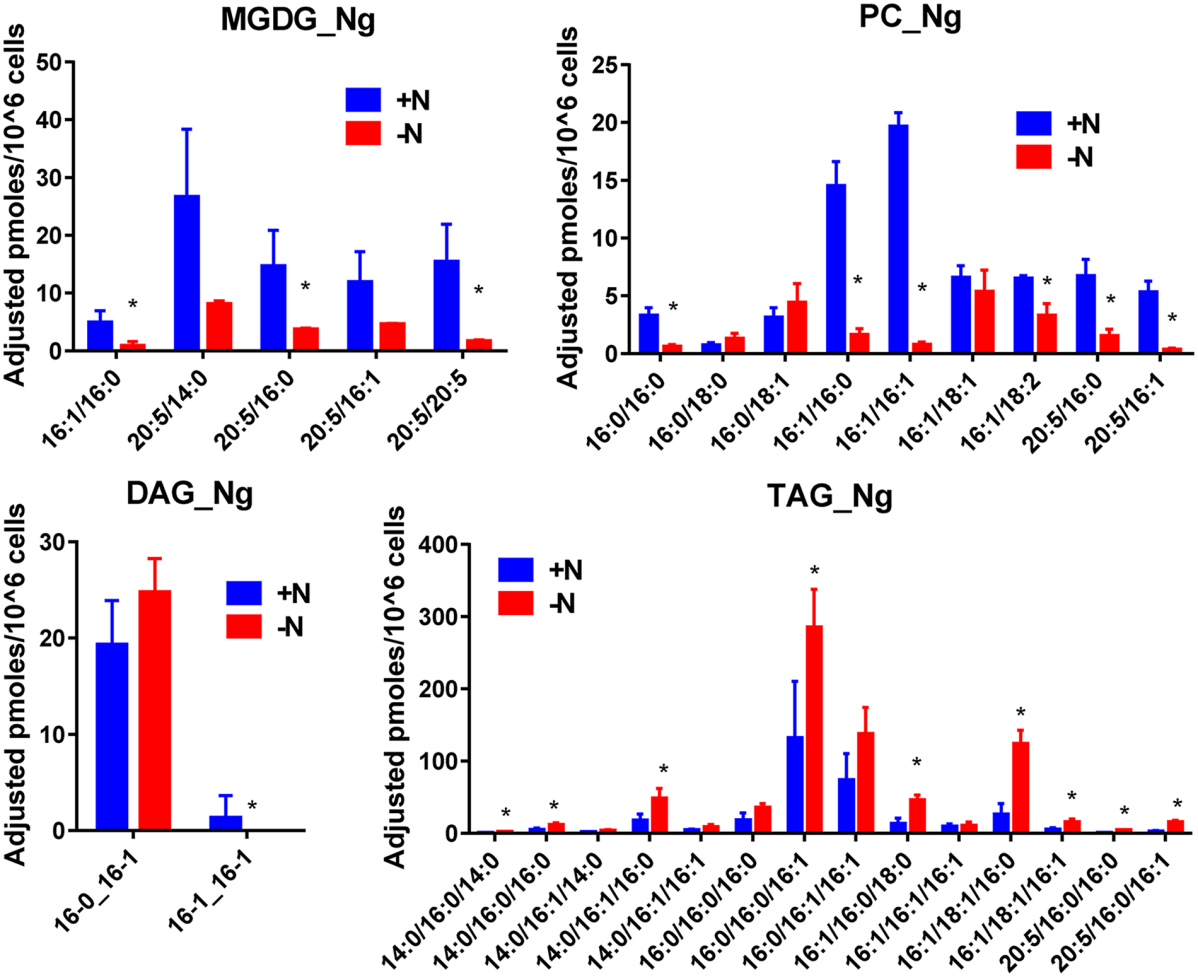
Impact of nitrogen starvation on the amount of the various molecular species constituting MGDG, PC, DAG and TAG in N.g.. Quantification were made by the LC-MS/MS method. Values are the average ± SD of three biological repeats. Significant differences (p ≤ 0.05) are indicated by an asterisk. Significant differences (P < 0.05) are shown by an asterisk and were calculated by an unpaired multiple t test using GraphPad Prism software.

**Fig 8:**
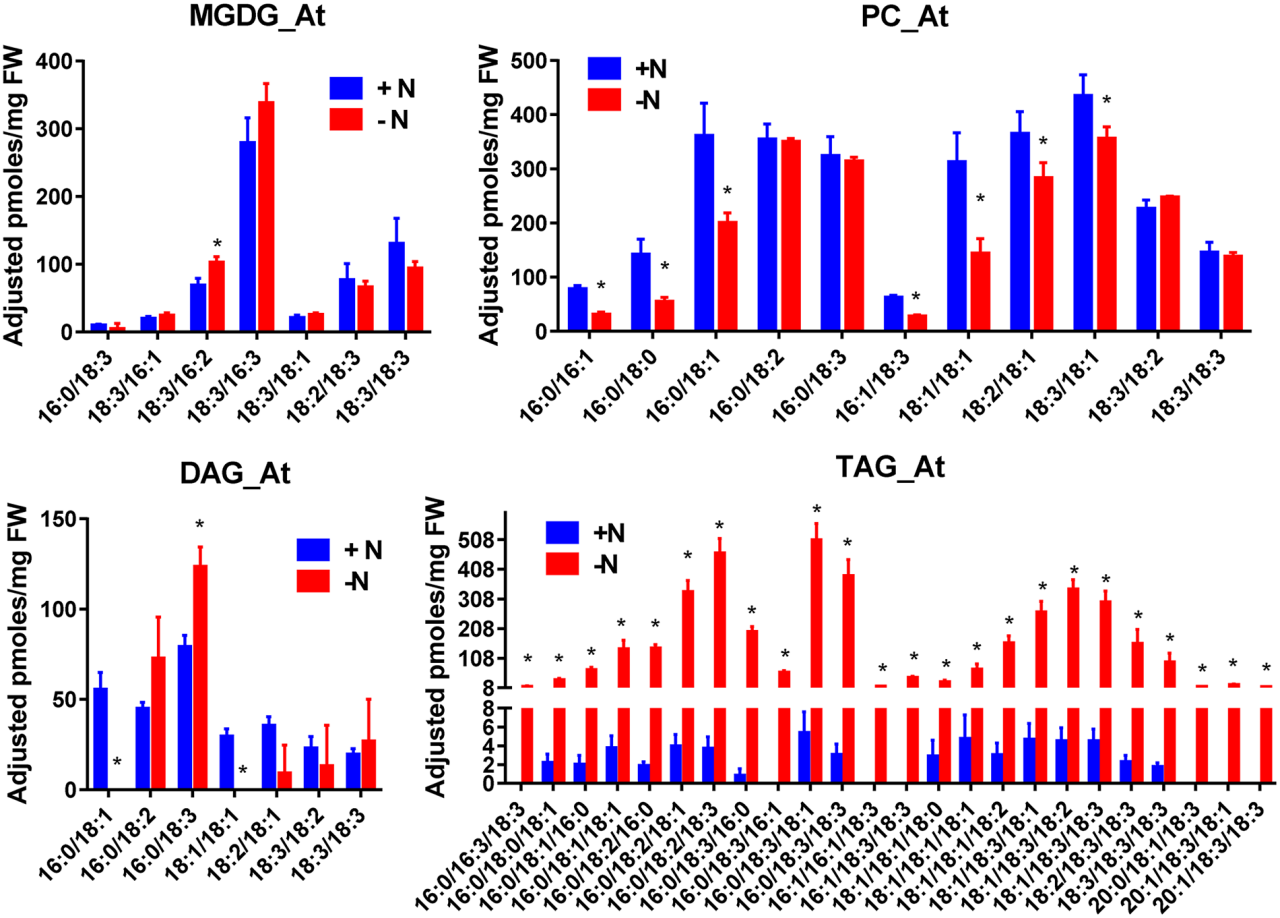
Impact of nitrogen starvation on the amount of the various molecular species constituting MGDG, PC, DAG and TAG in A.t.. Quantification were made by the LC-MS/MS method. Values are the average ± SD of three biological repeats. Significant differences (p ≤ 0.05) are indicated by an asterisk. Significant differences (P < 0.05) are shown by an asterisk and were calculated by an unpaired multiple t test using GraphPad Prism software.

Changes of the individual molecular species are shown in Figs 6 to 8. In P.t. (Fig 6), nitrogen starvation induced a decrease of most MGDG species. However, species containing 16:0 and 16:1 decreased less than those containing 20:5, with the noticeable exception of the 20:5/16:4. In fact, the amount of the species containing 20:5 decreased by a factor of 30 except for the 20:5/16:4 which remained constant and appeared to be the major MGDG species after seven days of nitrogen starvation.

This suggests that this particular species was less metabolized than the other ones in P.t.. Altogether the amount of 20:5 present in MGDG decreased about ten times, indicating that this long unsaturated FA was less present in plastids compared to 16:0 and 16:1. This strongly affected the distribution profile of the MGDG molecular species, as shown in S2 Fig. In contrast, the amount of PC molecules harboring 16:0 and 16:1 decreased more than those containing 20:5, suggesting that incorporation of 20:5 was favored in PC during nitrogen deprivation. FA distribution in DAG, essentially 16C, did not change much except for a slight increase of 20:5, whereas TAG species containing 16:0 and 16:1 increased most. Taken as a whole, these changes in the distribution of the molecular species in P.t. suggest that the newly synthesized TAG essentially originated from de novo synthesis of DAG rather than the recycling of membrane polar lipids.

In N.g. (Fig 7), there are fewer MGDG and PC molecular species than in P.t.. All main MGDG species contain 20:5 and they were lowered by a factor of 3–4 after seven days of starvation.

However, their distribution (%) remained roughly the same (S3 Fig), except for the 20:5/20:5, which decreased by a factor of two. In contrast with P.t., there is only few PC species containing 20:5 and more containing 16C and 18C. Interestingly, PC species containing 18:1 decreased less than other species during nitrogen deprivation. As a result, the 16:0/18:1 and 16:1/18:1 PC species appeared major in nitrogen starved cells (S3 Fig), which was not the case in control cells. As for P.t., DAG composition did not change much and displayed mainly 16:0 and 16:1. The effect of a nitrogen starvation on TAG resulted in an increase of the species containing 16C, but also of those containing 18:1, in particular the 16:0/18:1/16:1. Since we only detected 18:1 in PC, it is possible that PC also contributed to TAG synthesis, as already postulated in higher plants [9, 15].

In A.t. (Fig 8), the amount and the composition of MGDG remained constant after three days of nitrogen starvation.

The total amount of PC declined by about 25%, mainly because of the decrease of the species containing 18:0 or 18:1. In contrast, species containing 18:2 or 18:3 were almost not affected. DAG composition appeared also to be enriched in species containing 18:3, and poorer in species containing 18:1. In TAG, all the species increased upon starvation, but the species having 18:1 increased less than those having 18:3. This is more clearly seen when plotting the distribution (%) of these species (S4 Fig). In particular, the distribution of the species 16:0/18:3/X increased most during nitrogen starvation. Since 16:0/18:2 and 16:0/18:3 are the main DAG and PC species, it is possible that part of the newly synthesized TAG occurred via a PC to DAG to TAG pathway, as suggested previously [15, 47].

To summarize, a nitrogen deficiency in all three organisms induces an increase in TAG cell content and an arrest of cell division, but the pathways producing TAG seem to be organism-dependent. The TAG accumulation could be explain by the fact that photosynthesis is not totally abolished at the beginning of the nitrogen starvation whereas DNA and protein synthesis were strongly decreased, thereby blocking the cell division. TAG accumulation could be a way to store organic carbon produced during this time but the mechanisms involved remain to be unraveled.

## Conclusion

The main goal of this work is to point out how much a lipid composition can be different depending on the analytical method used. Biases linked to MS-based methods are well recognized in the scientific community, but often forgotten. There is probably no perfect method to measure the lipid composition in plants and microalgae, and this work is an attempt to reconcile these two main techniques because we believe it is important to be able to compare recent and older results, whatever the technology used.

Absolute quantification of lipids by ESI-MS/MS is difficult without a complete set of authentic ^13^C-labeled standards. The formation of ions in the ESI source is very dependent on the nature and chemical structure of the molecule. The use as internal standards of lipid species, which are not present in natural samples such as those having two saturated FA, cannot correct biases in some class of lipids such as MGDG or PC. We have no clear explanation for this, but it is possible that the presence of two saturated FA such as 18:0 impacts differently the formation of ions in the ESI source compared to a mixture of unsaturated FA. To circumvent this problem, we systematically run in tandem with the unknown sample a lipid extract (QC), previously quantified by TLC plus GC-FID methods and having a FA composition more or less similar to the one to be analyzed. Indeed, when quantification were made using QC as a quantitative reference, the lipid distribution data determined by ESI-MS/MS were similar to those obtained from the TLC plus GC-FID method. In each class, the amount of lipid determined with the QC method is the sum of the various molecular species present in this particular class. Because the nature of the FA may also impact the number of ions formed in the ESI source, we cannot ascertain the absolute quantification of each molecular species. However, the FA profiles recalculated from LC-MS/MS analyses for each class of lipid and compared to those directly obtained by GC-FID were roughly similar, at least for the most representative FA. This supports that there was no major bias in the estimation of the main molecular species present in each class of glycerolipids.

We used this approach to compare the impact of a nitrogen deficiency on the lipid profiles of three different types of cells. In all cases, nitrogen starvation was associated with a consistent strong increase of TAG, which represented in this situation 80% (microalgae) and 35% (Arabidopsis cells) of the total amount of glycerolipids. In the three types of cells, there was an increase of the total content of 16:0, indicative of de novo FA synthesis. Also, we observed in the three types of cells a decrease of membrane lipids, thus suggesting a reduction of the membrane network. In microalgae, thylakoids are the main membrane network whereas non-plastid membranes are more important in Arabidopsis cell cultures [28]. This might explain why lipids from plastids (MGDG, DGDG, SQDG, PG) decreased more significantly in microalgae, whereas phospholipids (PC, PE, PI, PG, DPG) decreased more significantly in A.t.. However, the impacts on individual membrane lipids appeared different from one type of cell to another, which makes difficult to point out a general rule about TAG accumulation that could be common to these organisms. In A.t., DAG and PC showed similar variations, in particular the 16:0/18:2 and the 16:0/18:3 species, and it seems likely that these DAG skeletons were at the origin of some of the newly synthesized TAG, thus providing a link between PC, DAG and TAG. In N.g., it is also possible that PC contributed to TAG synthesis since 18:1, only detected in PC, also increased in TAG during nitrogen starvation. There is no evidence that a same filiation occurred in P.t., where de novo synthesis of TAG appeared to be the main pathway as judged by the stronger increase of the species containing 16:0 and 16:1.

In conclusion, the presented use of a QC allows to benefit from both quantification accuracy and high throughput, two conditions required for glycerolipid profiling and analyses of large amounts of samples such as analyses of large collection of mutants, species variants in natural communities, or phenotype variations in multiple environment conditions.

## Supporting information

S1 Table Analytical parameters and MRM values used to identify the different glycerolipids present in *Phaeodactylum tricornutum*, *Nannochloropsis gaditana* and *Arabidopsis thaliana*. (XLSX)

S2 Table Positional distribution of fatty acids, and molecular species found in each glycerolipid classes from *Nannochloropsis gaditana*. Only molecules that represent more than 1% of all the species present in the class were indicated. The values represent the relative abundance within the class, as determined with the MS linear trap (see Material and methods). Major molecular species of a given lipid class are shown in bold characters. The asterisk indicates where the *sn*-1 and *sn*-2 positions could not be discriminated.
(DOCX)

S3 Table Positional distribution of fatty acids, and molecular species found in each glycerolipid classes from *Arabidopsis thaliana* cell cultures. Only molecules that represent more than 1% of all the species present in the class were indicated. The values represent the relative abundance within the class, as determined with the MS linear trap (see Material and methods). Major molecular species of a given lipid class are shown in bold characters. The asterisk indicates where the *sn*-1 and *sn*-2 positions could not be discriminate.
(DOCX)

S1 Fig Quantification and response linearity using LC-MS/MS. A) the total number of pmoles of lipid recovered from a given number injected was estimated using either the internal standards (LC-MS/MS-Stds) or external standards (LC-MS/MS-QC) methods. B) the LC-MS/MS-QC method is used for quantification of the main classes of glycerolipids present in P.t., N.g. and A.t.. The initial amount of lipid was estimated by GC-FID and corresponds to total FA. The amount of FA recovered was calculated by multiplying by a factor of 2 each glycerolipid molecules except TAG that were multiplied by a factor of 3. Values are the average ± SD of three technical repeats.
(TIF)

S2 Fig Impact of nitrogen starvation on the distribution (%) of the various molecular species constituting MGDG, PC, DAG and TAG in P.t.. Quantification were made by the LC-MS/MS method. Values are the average ± SD of three biological repeats. Significant differences (p ≤ 0.05) are indicated by an asterisk.
(TIF)

S3 Fig Impact of nitrogen starvation on the distribution (%) of the various molecular species constituting MGDG, PC, DAG and TAG in N.g.. Quantification were made by the LC-MS/MS method. Values are the average ± SD of three biological repeats. Significant differences (p ≤ 0.05) are indicated by an asterisk.
(TIF)

S4 Fig Impact of nitrogen starvation on the distribution (%) of the various molecular species constituting MGDG, PC, DAG and TAG in A.t.. Quantification were made by the LC-MS/MS method. Values are the average ± SD of three biological repeats. Significant differences (p ≤ 0.05) are indicated by an asterisk.
(TIF)

## Abbreviations

DAG: diacylglycerol
FA: fatty acid
FAME: fatty acid methyl ester
FID: Flame ionization detector
LC: Liquid chromatography
MGDG: Monogalactosyldiacylglycerol
MS: mass spectrometry
PC: Phosphatidylcholine
QC: Qualified control
TAG: triacylglycerol
TLC: Thin Layer Chromatography
