# Correction: LC-MS/MS versus TLC plus GC methods: Consistency of glycerolipid and fatty acid profiles in microalgae and higher plant cells and effect of a nitrogen starvation

**DOI:** 10.1371/journal.pone.0206397

**Published:** 2018-10-22

**Authors:** Juliette Jouhet, Josselin Lupette, Olivier Clerc, Leonardo Magneschi, Mariette Bedhomme, Séverine Collin, Sylvaine Roy, Eric Maréchal, Fabrice Rébeillé

The following information is missing from the Competing Interests and Funding section: Total Refining Inc. provided support in the form of salary for SC but did not have any additional role in the study design, data collection and analysis, decision to publish, or preparation of the manuscript. The specific roles of this author are articulated in the ‘author contributions’ section. This does not alter our adherence to all *PLOS ONE* policies on sharing data and materials.

Additionally, the data underlying this study is omitted. The authors have provided the complete dataset below as S1 Data.

## Supporting information

S1 Data Supplementary dataset. This file includes supplementary data.
(XLSX)
