# Supplementary material for: LC-MS/MS versus TLC plus GC methods: Consistency of glycerolipid and fatty acid profiles in microalgae and higher plant cells and effect of a nitrogen starvation
Source: PLoS One. 2017 Aug 3;12(8):e0182423. doi: 10.1371/journal.pone.0182423 (PMC5542700; doi:10.1371/journal.pone.0182423)
Supplement: S3 Table — Only molecules that represent more than 1% of all the species present in the class were indicated. The values represent the relative abundance within the class, as determined with the MS linear trap (see Material and methods). Major molecular species of a given lipid class are shown in bold characters. The asterisk indicates where the sn-1 and sn-2 positions could not be discriminate. (DOCX) [file pone.0182423.s003.docx]

**Supplemental Table 3: Positional distribution of fatty acids, and molecular species found in each glycerolipid classes from *Arabidopsis thaliana* cell cultures.** Only molecules that represent more than 1% of all the species present in the class were indicated. The values represent the relative abundance within the class, as determined with the MS linear trap (see Material and Methods). Major molecular species of a given lipid class are shown in bold characters. The asterisk indicates where the *sn*-1 and *sn*-2 positions could not be discriminate.

| ***sn-1/sn-2*** | **SQDG** | **MGDG** | **DGDG** | **PG** | **PI** | **PS** | **PE** | **PC** | **PA** | **DAG** | **TAG** |
| --- | --- | --- | --- | --- | --- | --- | --- | --- | --- | --- | --- |
| 16:0/16:0 | 2.8 | 1.3 | 1 | 9 |  | **26** |  |  |  |  |  |
| 16:0/16:1 |  |  | 2 | 7 |  | 7 | 2.5 | 4 |  | 5 |  |
| 16:0/16:3 |  |  | 1.3 |  |  | 2 |  |  |  |  |  |
| 16:0/18:0 | 1.8 |  |  |  | 3 |  |  | 2 | 2 | 8 |  |
| 16:0/18:1 | 7 |  | 2.5* | 19* | 21 | 6 | 11 | 14 | 9 | **13** |  |
| 16:0/18:2 |  |  |  | 18 | 19 | 6 | 15 | 12 | 6 | 9 |  |
| 16:0/18:3 |  | 1 | 9 |  | **38** | 10 | **20** | **16** | 11 | **12** |  |
| 16:1/16:0 | 3.2 |  |  |  |  |  |  |  |  |  |  |
| 16:1/16:1 |  |  |  |  |  |  |  |  |  |  |  |
| 16:1/16:2 |  |  |  |  |  |  |  |  |  |  |  |
| 16:1/18:1 | 4 |  |  |  |  |  |  |  |  |  |  |
| 16:1/18:2 |  |  |  |  |  |  |  |  |  |  |  |
| 16:1/18:3 | 2* |  |  |  | 1.5 | 4 | 2 | 2 | 4* |  |  |
| 16:2/16:0 |  |  |  |  |  | 1.5 |  |  |  |  |  |
| 18:0/16:0 |  |  |  | 2.5 |  |  |  |  |  |  |  |
| 18:0/18:1 |  |  |  |  | 1 | 4 | 2 |  | 3 |  |  |
| 18:0/18:3 |  |  |  |  |  | 2.5 |  | 2 |  |  |  |
| 18:1/16:0 |  |  | 2.5* | 19* |  |  |  |  |  |  |  |
| 18:1/16:1 |  |  |  |  |  |  |  |  | 3 |  |  |
| 18:1/18:0 | 1 |  |  |  |  |  |  |  |  |  |  |
| 18:1/18:1 | 2 | 1 |  | 1 | 5 | 8 | 6 | 5 | 10 | 9 |  |
| 18:1/18:2 |  | 1.5* | 1.5 |  | 4 | 5 | 9 | 9* | 12 |  |  |
| 18:1/18:3 | 9* | 4* | 11 | 1.5* | 6 | 10 | 10 | **13*** | **20*** |  |  |
| 18:2/16:0 | 8 |  | 5 |  |  |  |  |  |  |  |  |
| 18:2/16:3 |  | 2.5 |  |  |  |  |  |  |  |  |  |
| 18:2/18:1 | 2 | 1.5* |  |  |  |  |  | 9* |  | 10 |  |
| 18:2/18:2 |  |  |  |  |  |  | 5 | 6 |  |  |  |
| 18:2/18:3 |  | 6 | 10 |  | 1.5* | 1.7 |  | 7* | 11* |  |  |
| 18:3/16:0 | **42** |  |  | **32** |  |  |  |  |  |  |  |
| 18:3/16:1 | 2* | 2.5 | 5 | 10 |  |  |  |  | 4* |  |  |
| 18:3/16:2 |  | 4 | 2.5 |  |  |  |  |  |  |  |  |
| 18:3/16:3 |  | **38** | 5 |  |  |  |  |  |  |  |  |
| 18:3/18:0 | 1 |  |  |  |  |  |  |  |  |  |  |
| 18:3/18:1 | 9* |  |  | 1.5* |  |  |  | **13*** | **20*** | **19** |  |
| 18:3/18:2 | 3.4 |  |  |  | 1.5* |  | 7 | 7* | 11* | 6 |  |
| 18:3/18:3 | 8.5 | **31** | **37** |  |  | 1 | 6 | 6 | 9 | 6 |  |
| ***sn-1/sn-2/sn-3*** |  |  |  |  |  |  |  |  |  |  |  |
| 16:0/16:3/18:3 |  |  |  |  |  |  |  |  |  |  | 3 |
| 16:0/18:0/18:1 |  |  |  |  |  |  |  |  |  |  | 3.5 |
| 16:0/18:1/16:0 |  |  |  |  |  |  |  |  |  |  | 3 |
| 16:0/18:1/18:1 |  |  |  |  |  |  |  |  |  |  | 4 |
| 16:0/18:1/18:2 |  |  |  |  |  |  |  |  |  |  |  |
| 16:0/18:2/16:0 |  |  |  |  |  |  |  |  |  |  | 3 |
| 16:0/18:2/18:1 |  |  |  |  |  |  |  |  |  |  | 4 |
| 16:0/18:3/16:0 |  |  |  |  |  |  |  |  |  |  | 3 |
| 16:0/18:3/16:1 |  |  |  |  |  |  |  |  |  |  | 2.5 |
| 16:0/18:3/18:1 |  |  |  |  |  |  |  |  |  |  | **8** |
| 16:0/18:3/18:2 |  |  |  |  |  |  |  |  |  |  | 6 |
| 16:0/18:3/18:3 |  |  |  |  |  |  |  |  |  |  | 4 |
| 16:1/16:1/18:3 |  |  |  |  |  |  |  |  |  |  | 4 |
| 16:1/18:3/18:3 |  |  |  |  |  |  |  |  |  |  | 3.5 |
| 18:1/18:1/18:0 |  |  |  |  |  |  |  |  |  |  | 4 |
| 18:1/18:1/18:1 |  |  |  |  |  |  |  |  |  |  | 4 |
| 18:1/18:1/18:2 |  |  |  |  |  |  |  |  |  |  | 5 |
| 18:1/18:3/18:1 |  |  |  |  |  |  |  |  |  |  | **7** |
| 18:1/18:3/18:2 |  |  |  |  |  |  |  |  |  |  | **7** |
| 18:1/18:3/18:3 |  |  |  |  |  |  |  |  |  |  | **7** |
| 18:2/18:3/18:3 |  |  |  |  |  |  |  |  |  |  | 5 |
| 18:3/18:3/18:3 |  |  |  |  |  |  |  |  |  |  | 5 |
| 20:0/18:1/18:3 |  |  |  |  |  |  |  |  |  |  | 1.5 |
| 20:0/18:3/18:3 |  |  |  |  |  |  |  |  |  |  | 2 |
| 20:1/18:3/18:1 |  |  |  |  |  |  |  |  |  |  | 2 |
| 20:1/18:3/18:3 |  |  |  |  |  |  |  |  |  |  | 2.5 |
