# Supplementary figures and images for: LC-MS/MS versus TLC plus GC methods: Consistency of glycerolipid and fatty acid profiles in microalgae and higher plant cells and effect of a nitrogen starvation
Source: PLoS One. 2017 Aug 3;12(8):e0182423. doi: 10.1371/journal.pone.0182423 (PMC5542700; doi:10.1371/journal.pone.0182423)

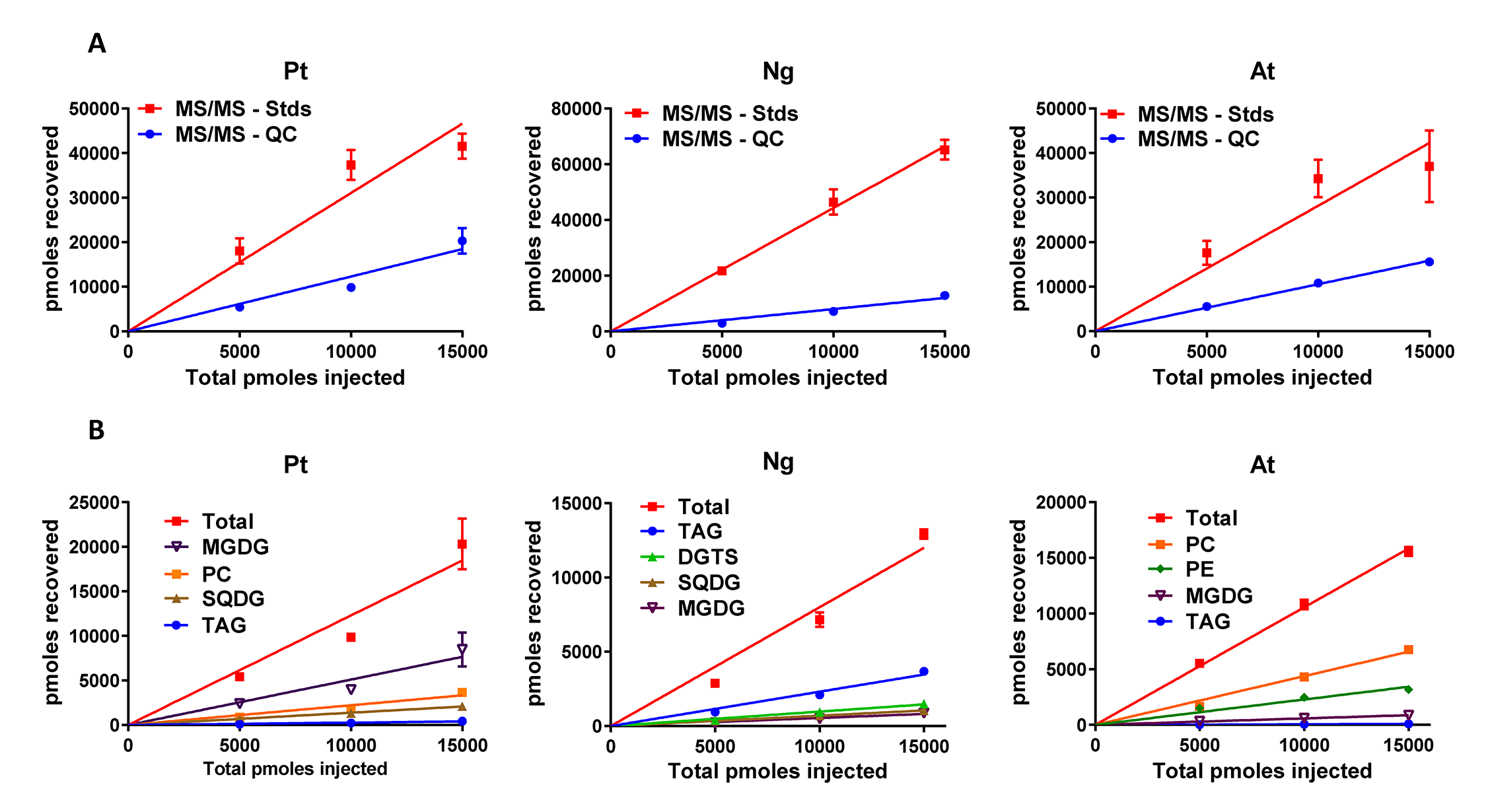

Supplement: S1 Fig — A) the total number of pmoles of lipid recovered from a given number injected was estimated using either the internal standards (LC-MS/MS-Stds) or external standards (LC-MS/MS-QC) methods. B) the LC-MS/MS-QC method is used for quantification of the main classes of glycerolipids present in P.t., N.g. and A.t.. The initial amount of lipid was estimated by GC-FID and corresponds to total FA. The amount of FA recovered was calculated by multiplying by a factor of 2 each glycerolipid molecules except TAG that were multiplied by a factor of 3. Values are the average ± SD of three technical repeats. (TIF) [file pone.0182423.s004.tif]

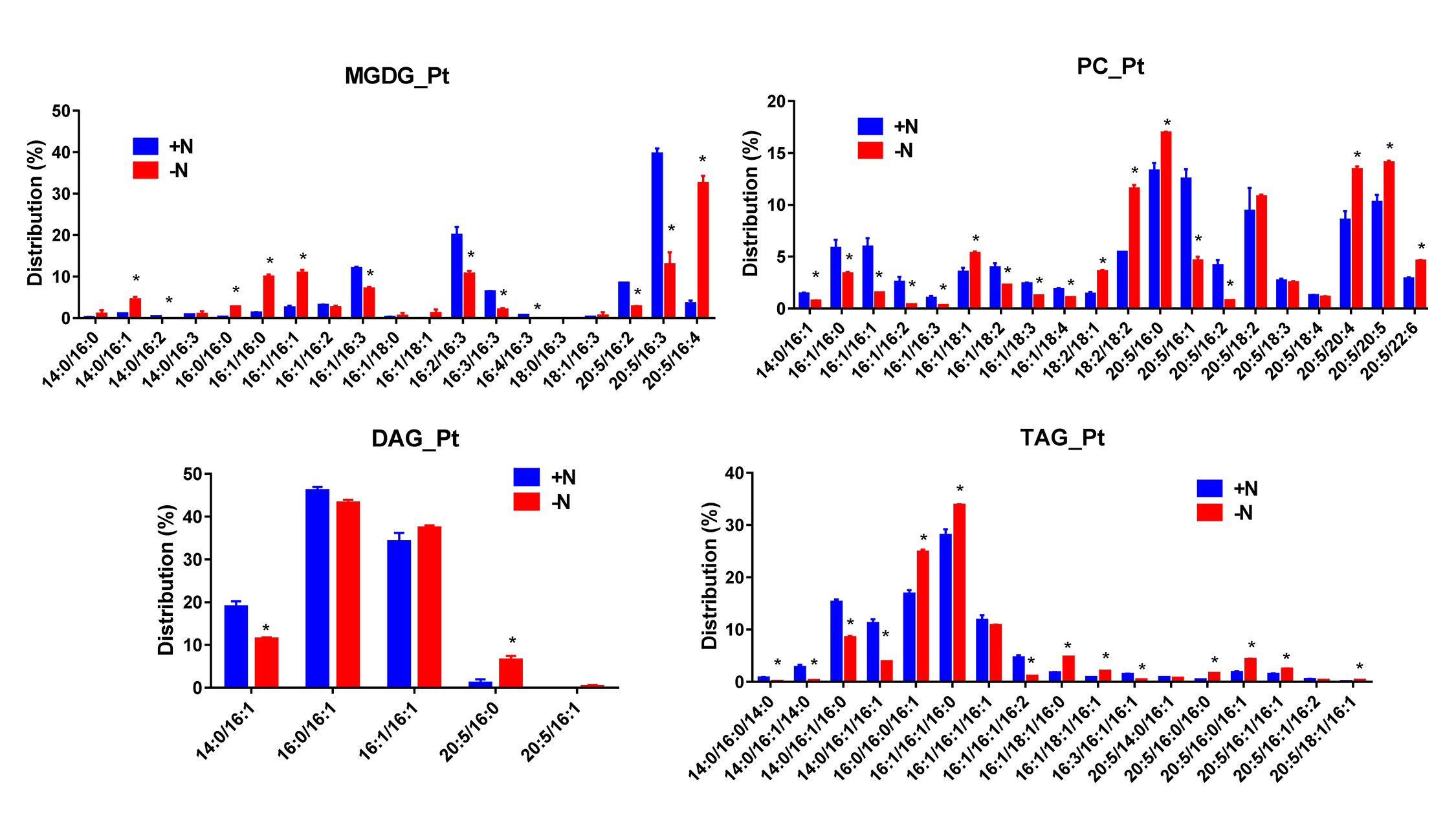

Supplement: S2 Fig — Quantification were made by the LC-MS/MS method. Values are the average ± SD of three biological repeats. Significant differences (p ≤ 0.05) are indicated by an asterisk. (TIF) [file pone.0182423.s005.tif]

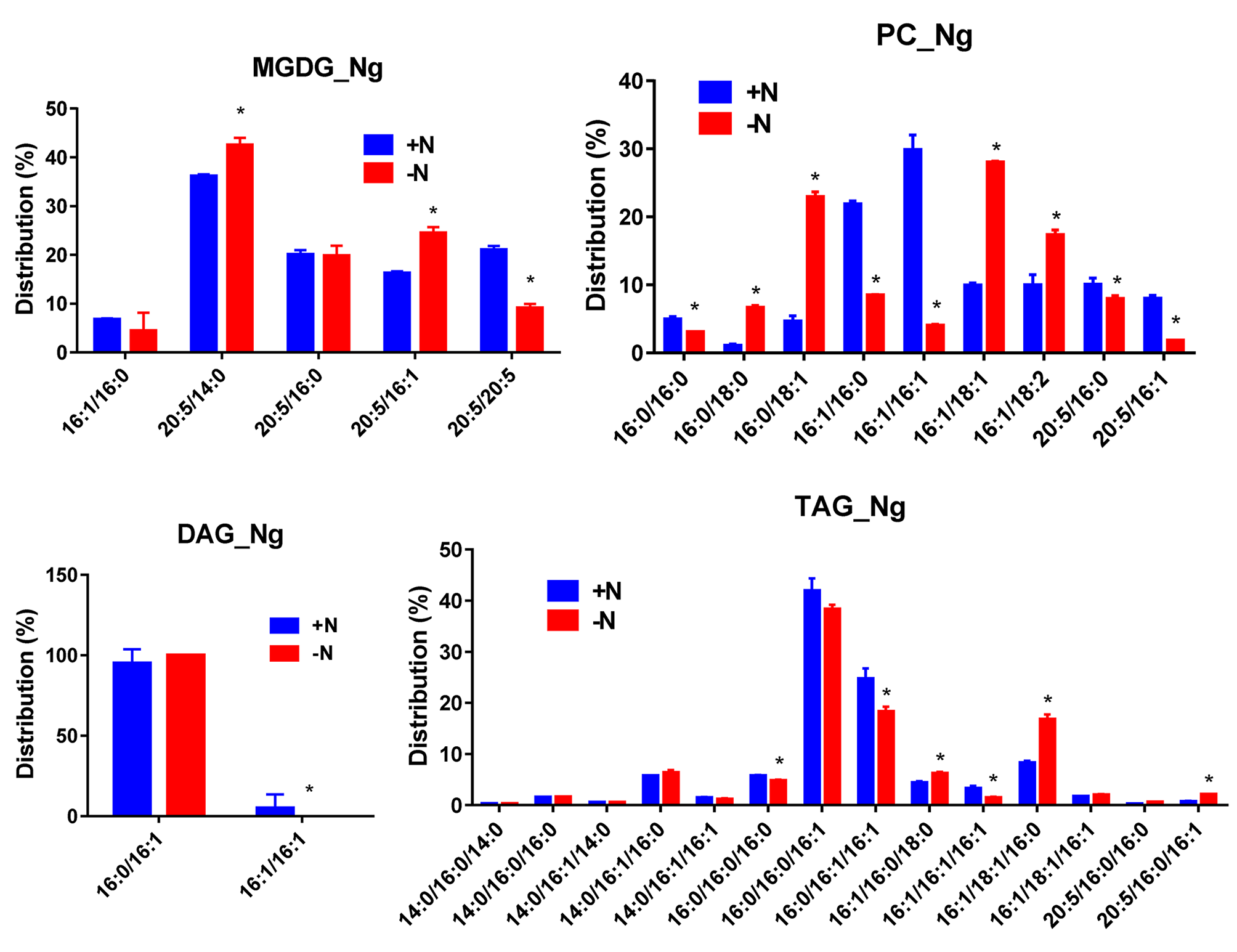

Supplement: S3 Fig — Quantification were made by the LC-MS/MS method. Values are the average ± SD of three biological repeats. Significant differences (p ≤ 0.05) are indicated by an asterisk. (TIF) [file pone.0182423.s006.tif]

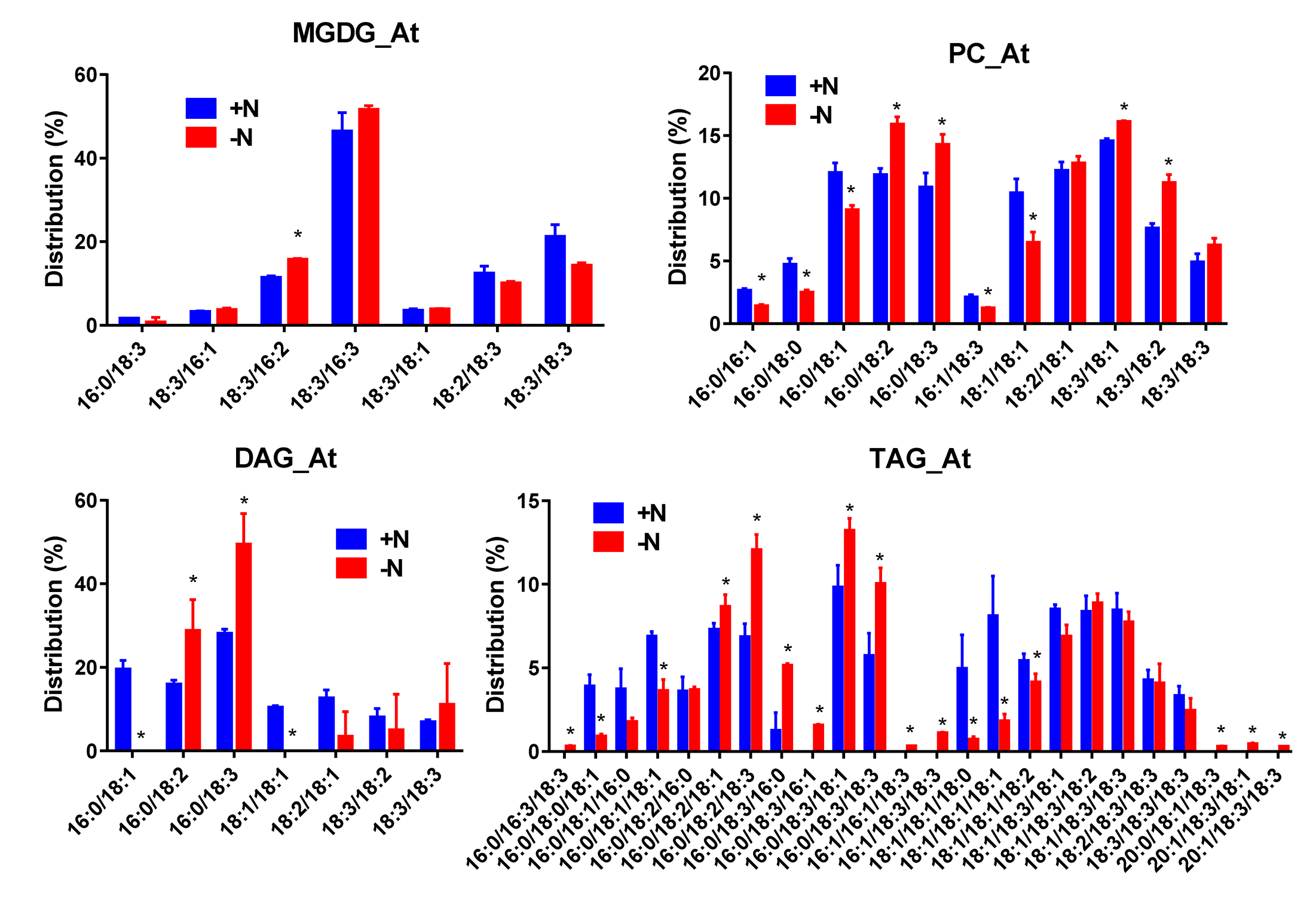

Supplement: S4 Fig — Quantification were made by the LC-MS/MS method. Values are the average ± SD of three biological repeats. Significant differences (p ≤ 0.05) are indicated by an asterisk. (TIF) [file pone.0182423.s007.tif]
